# The New Physiological Buildings at Cardiff

**Published:** 1915-08-21

**Authors:** 


### THE NEW PHYSIOLOGICAL BUILDINGS AT CARDIFF.

### The Question of a Treasury Grant.

ON the occasion of laying the foundation stone of the new Physiological Buildings at Cardiff on the 12th inst., Sir William James Thomas remarked that to the father of Lord Aberdare, the President, was due, as much as to any other man, the establishment of the University College of South Wales and Monmouthshire. Moreover, it was also his great work as chairman of a Departmental Committee of Inquiry, appointed on August 25, 1880, exactly thirty-five years ago, that we are indebted to what may be called without exaggeration the most important document ever published in connection with education in Wales; indeed, it is the "Charter of Education of Modern Wales." We are also indebted to Lord Pontypridd for laying the foundation stone of what we all hope to be "The Welsh National School of Medicine," for which Colonel Bruce Vaughan had worked so hard. They were proud that Sir William Osler had been able to be present to give them his counsel and wise advice on this occasion.

Sir W. J. Thomas then added:

I have been, as you all know, greatly interested for many years in the work of our hospitals; and what a testimony to their work is to be found in the fact that all the resources of science, medicine, surgery, and nursing are placed at the soldier's service on campaign even more insistently than they were when he was a civilian. When Colonel Bruce Vaughan placed the claims of the Medical School before me, I was easily convinced of the necessity of such a school. I willingly promised to perform a share in setting up this school by providing the necessary buildings, for I had been convinced that, unless the several departments of a modern school were well housed and equipped, we should not attract men who were sufficiently highly qualified to make this a leading school. I have left much for others to do, and I hope others will endow scholarships and provide other great prizes, so that there shall be as many attractions for students here at Cardiff as anywhere among the medical schools. Our present school loses its most distinguished students; they become attached to London and even Colonial schools in consequence of Cardiff not possessing a complete school. Here is another great opportunity to retain the services and future distinctions of these students for Wales by endowing research, thus enabling the greatest brains Wales produces to spend all their days in research that may not bring immediate results, but whose reward would come in the improved health and happiness of future generations.

Another great inducement held out to me, and I must confess it largely influenced my decision, was that the offer of the provision of buildings for a complete, up-to-date school would induce the Treasury to make an adequate grant for its maintenance. The belief that this would be so was expressed by Mr. Lloyd George in his speech to the deputation, when he said that "he would advise His Majesty's Government to make a substantial contribution to the appeal that had been so earnestly placed before him."

There is just one other reason present in my mind. It is that we should establish in Wales at least one full School of Medicine, so that parents of boys, who are not in a position to send them to London or elsewhere for the latter half of their training, should be able in the immediate future to provide facilities here in Cardiff for entering the medical profession which the more fortunate students of England, Scotland, and Ireland have enjoyed for centuries.

Mr. Lloyd George expressed the same desire when replying to the deputation on February 18, 1914. He said: "The cost of medical education was almost prohibitive, except for people who could spend large sums upon the education of their children, and he hoped that the establishment of the school at Cardiff would make it easier for children of parents of small incomes to enter that noble profession."

If this wise suggestion be carried out, may we not find encouragement in the humble origin of Franklin, Faraday, and Pasteur; and may we not expect our country to produce many men like Louis Pasteur's father, a private soldier of the First Empire, a hard-working tanner? Let all Welsh parents take Pasteur's gratitude to his father to heart. If, by others following my example, a wise and generous co-operation by the State is induced, the day of the inauguration of a complete Medical School for Wales will be the proudest of my life.

